# Spatial differentiation and determinants of COVID-19 in Indonesia

**DOI:** 10.1186/s12889-022-13316-4

**Published:** 2022-05-23

**Authors:** Millary Agung Widiawaty, Kuok Choy Lam, Moh Dede, Nur Hakimah Asnawi

**Affiliations:** 1 Faculty of Social Sciences Education (FPIPS), Universitas Pendidikan Indonesia, Jln. Dr. Setiabudho no. 299, Bandung City, West Java 40154 Indonesia; 2 National Research and Innovation Agency of Indonesia (BRIN), Jln. Kuningan Barat, Mampang Prapatan, Jakarta, 12710 Indonesia; 3 Geography Program, Centre for Research in Development, Social and Environment, Faculty of Social Sciences and Humanities, Universiti Kebangsaan Malaysia, Bangi, 43600 Selangor Malaysia; 4 Center for Environment and Sustainability Science, Universitas Padjadjaran, Bandung City, West Java 40132 Indonesia

**Keywords:** COVID-19, Socioenvironmental factors, Spatial interaction

## Abstract

**Background:**

The spread of the coronavirus disease 2019 (COVID-19) has increasingly agonized daily lives worldwide. As an archipelagic country, Indonesia has various physical and social environments, which implies that each region has a different response to the pandemic. This study aims to analyze the spatial differentiation of COVID-19 in Indonesia and its interactions with socioenvironmental factors.

**Methods:**

The socioenvironmental factors include seven variables, namely, the internet development index, literacy index, average temperature, urban index, poverty rate, population density (PD) and commuter worker (CW) rate. The multiple linear regression (MLR) and geographically weighted regression (GWR) models are used to analyze the impact of the socioenvironmental factors on COVID-19 cases. COVID-19 data is obtained from the Indonesian Ministry of Health until November 30th 2020.

**Results:**

Results show that the COVID-19 cases in Indonesia are concentrated in Java, which is a densely populated area with high urbanization and industrialization. The other provinces with numerous confirmed COVID-19 cases include South Sulawesi, Bali, and North Sumatra. This study shows that the socioenvironmental factors, simultaneously, influence the increasing of confirmed COVID-19 cases in the 34 provinces of Indonesia. Spatial interactions between the variables in the GWR model are relatively better than those between the variables in the MLR model. The highest spatial tendency is observed outside Java, such as in East Nusa Tenggara, West Nusa Tenggara, and Bali.

**Conclusion:**

Priority for mitigation and outbreak management should be high in areas with high PD, urbanized spaces, and CW.

## Background

The year 2019 ended with the outbreak of the novel coronavirus or severe acute respiratory syndrome coronavirus-2 (SARS-CoV-2) in Wuhan, the People’s Republic of China. This virus was initially called 2019-nCoV but later renamed by the World Health Organization (WHO) as SARS-CoV-2, causing the coronavirus disease (COVID-19) and various symptoms similar to those of SARS, which previously plagued the world [1–3]. People infected by COVID-19 often potentially have associated long term symptoms, know as long COVID-19, such as fatigue, anxiety, breathlessness, dizziness, depression, sleep difficulty, palpitations among others [4, 5]. At the end of 2020, COVID-19 affected more than 80 million people worldwide and 1.8 million deaths (with a case fatality rate [CFR] of 2.2%). The spread of this disease is higher than that of the Spanish flu in the twentieth century. Currently, the spread of COVID-19 is increasing once again owing to the virus mutation SARS-CoV-2 VUI 202012/01 (Variant Under Investigation), which is highly contagious [6]. Within the global community, SARS-CoV-2 is known as the COVID-19 virus, which belongs to the family of viruses causing symptoms such as fever, cough, sore throat, headache, vomiting and others [7, 8]. The virus attacks the respiratory system of people of all ages, thereby endangering individuals with other types of diseases (comorbidities) particularly to those with cardiovascular diseases, obesity, diabetes, and hypertension [9, 10]. Existing health conditions remain the main factor aggravating COVID-19 in patients. In response, a rapid spread of COVID-19 has enforced the governments worldwide to implement a strict lockdown or movement control in their country to curb this virus spreading. Consequently, most of the world economic activities were pending and led to detrimental socioeconomic effects [11]. In contrast, the reduction of worldwide socioeconomic activities has brought somewhat positive impacts on natural environment [12, 13].

Statistically, the ASEAN countries had more than 1.5 million confirmed cases by 31 December 2020. COVID-19 in this region spread mainly in Indonesia, with more than 700 thousand confirmed cases, 600 thousand recovered cases and 22 thousand deaths. Indonesia accounts for 48.82% of the total confirmed COVID-19 cases in the ASEAN countries. In terms of the CFR, Indonesia leads with 3.0% compared with Vietnam (2.4%), Malaysia (0.42%) and Thailand (0.9%) [14]. COVID-19 has spread globally, including in Indonesia. From the onset of the increasing number of confirmed cases, the daily COVID-19 cases in Indonesia have yet to show signs of decreasing. In terms of environmental conditions for the spread of COVID-19, Indonesia’s physical environment is relatively unsuitable for the virus, which is susceptible to high temperatures and ultraviolet exposure [15]. Thus, COVID-19 cases in the country should have been controlled easily, such as those in Vietnam, Malaysia and Thailand [16]. Indonesia is an archipelagic country with a tropical climate that receives sunlight nearly all year round. However, these conditions do not guarantee low numbers of COVID-19 cases. The spread of COVID-19 in Indonesia is a cause of concern and should be addressed through the implementation of appropriate policies, especially physical-social restrictions, increasing vaccination coverage, and tracing of the viral transmission. Policies in Indonesia for handling COVID-19 are lacking, as existing ones seems to be focused on distributing social subsidy [17]. To establish effective policies, stakeholders should conduct COVID-19 assessments, with physical and social environment conditions as main inputs for decision making. The government and the people must not be lulled by the success of the physical and social restrictions resulted from the implementation of emergency public activity restrictions and large-scale social restrictions programme [18], because the virus is always mutating and can become more infectious. The physical and social restriction’s policy proven to reduce the transmission rate of COVID-19, apart from being complemented by the mass vaccination programme and natural antibodies developed by the Covid-19 patients who recovered from the infections [19].

However, increased exposure to COVID-19 and the acceleration or support of its spread can be caused by not only individuals’ health conditions but also areas’ environmental and social factors. Environmental factors such as climate, humidity and ultraviolet radiation play a role in the spread of COVID-19 [20, 21], a long-duration of exposure with the infected person was the most significant risk factor of the COVID-19 disease spreads [22]. In addition, people’s activities can affect the spread of COVID-19. Social factors include population size and population density (PD) [23]. Apart from the physical environment, socioeconomic conditions can influence the spread of the disease. Previous research showed that socioenvironmental conditions have a strong link to high numbers of COVID-19 cases [24]. Busy and crowded places, such as markets, tourist destinations, offices, and schools, have closed temporarily, thereby resulting in risks of national economic recession and increasing unemployment, which may lead to poverty [25].

The number of confirmed cases is also closely related to PD, with densely populated urban areas causing the transmission rate to increase [26]. The community literacy level is another socioenvironmental factor affecting COVID-19 transmission. Presently, certain groups of people believe that the pandemic is part of a global conspiracy or ploy to increase health-related businesses, resulting in their refusal to follow health protocols. This refusal is potentially dangerous, especially in certain age groups, who may be infected by SARS-CoV-2 without exhibiting any symptoms, thereby acting as spreading agents to their families and others in their surroundings [27, 28]. This phenomenon is common among commuter workers (CWs) who do not comply with health protocols, such as maintaining distance, washing hands with soap, or using hand sanitizers and wearing masks [29]. Based on these facts, this study aims to analyze the spatial interactions between socioenvironmental factors and COVID-19 in Indonesia.

## Methods

### Research location

**Fig. 1:**
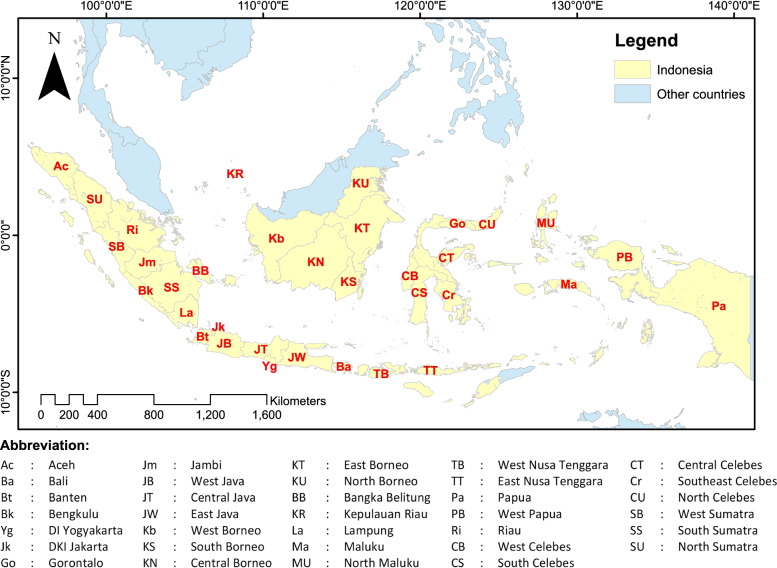
Research location of 34 provinces as the analysis unit

This study used provincial regions as the analysis unit. Indonesia has 34 provinces in accordance with the current first-level administrative division (Fig. 1). In Indonesia, the number of confirmed positive COVID-19 cases is increasing, with varying rates in the different provinces. The regional autonomy regulations in the country make each province responsible for handling COVID-19 cases through a task force, in which serious violations are the responsibility of the governor and regent/mayor, as stated in the Instruction of Minister of Home Affairs Number 01 Year 2020 [30]. Despite the poor data synchronization in the first months of the COVID-19 pandemic, emergency communication for disaster management was revamped in November 2020, which reduced data differences between the different government levels [31]. In the final COVID-19 recording for November 2020, Indonesia had 538,883 confirmed cases, including 450,518 recoveries and 16,945 deaths [32]. These statistics marked Indonesia as the country with the highest number of deaths due to COVID-19 in Asia, thereby causing the prohibition of Indonesians from entering other countries. Since the announcement of the first confirmed cases in Indonesia, the COVID-19 curve has yet to slope [33], and the end of the first wave only came by middle of 2021.

### Data acquisition and variables

Physical and socioenvironmental conditions are known to have a relationship with the spread of diseases (endemic, epidemic or pandemic). Physical environment factors, especially temperature and humidity, can affect the transmission of viruses and other microorganisms, and numerous diseases in tropical environments, such as dengue, malaria, tuberculosis, avian influenza and so on, have changed from a pandemic or an epidemic to being endemic [34]. Migration and intensive interactions among human populations also cause the transmission of diseases, especially in developing countries in the process of enhancing the quality of their human resources through education and health. In response to this phenomenon, many countries established tropical disease research centers, and scientists developed disease spread and outbreak models to address health, economic and security crises [35].

This research used reliable secondary data from authorized and credible Indonesian government agencies, such as the COVID-19 Task Force; Indonesian Ministry of Health; Indonesian Ministry of Manpower and Transmigration; National Development Planning Agency (Bappenas); Bureau of Meteorology, Climatology and Geophysics (BMKG); and the Indonesian Statistics Agency (BPS). The data were classified as open data, which can be reused for research purposes and decision making and accessed online through the official website of the providers [36]. The socioenvironmental factors used in this study included seven independent variables, namely, the Internet development index (IDI; X_1_), literacy index (LI; X_2_), average temperature (AT; X_3_), urban index (UI; X_4_), poverty rate (PR; X_5_), population density (PD; X_6_) and commuter workers rate (CW; X_7_). The socioenvironmental factor data were checked for errors, aggregated at the provincial level, and integrated in the GIS spatial database. The standardized administrative boundaries established by the National Geospatial Information Agency were used as the base map to facilitate the integration of the spatial database. The selection of the variables was based on the increasing threat of disasters and vulnerability in Indonesia owing to COVID-19. The dependent variable in this study was confirmed COVID-19 confirmed cases (Cov; Y) based on the polymerase chain reaction swab test results of SARS-COV-2 carriers in accordance with the confirmation of a government-appointed minimum biosafety level-2 laboratory [37]. Data updating on COVID-19 cases in Indonesia is conducted daily at 3:00–4:00 PM (Western Indonesian Time), which can be viewed via broadcasts on national television or the COVID-19 Task Force webpage.

### Data analysis

Disease spread models can be analyzed using mathematical models requiring scientists to understand the driving factors [38]. Multivariate models for disease spread analysis include the multiple linear regression (MLR) and geographically weighted regression (GWR) models. The MLR model can define independent variables triggering disease transmission and infection and is flexible and robust for disease spread spatial analysis [39, 40]. The MLR model has other advantages, such as its long developmental history and use in the transmission and mortality analyses of various infectious diseases, such as zika, tuberculosis, dengue, meningococcal, influenza and COVID-19 [41, 42]. Meanwhile, the GWR model is developed from regression models involving spatial variability as weights for compiling regression parameters [36]. The GWR model is used in numerous countries as an analytical tool for the spread of malaria, tuberculosis, and COVID-19. Epidemiological studies typically use both models to determine whether location has an effect on the research variables [42–44]. The MLR and GWR models have several similarities. Specifically, they both use continuous data and are bound to linear regression assumptions [45, 46].

A quantitative approach was used in this research to reveal the spatial interactions between the socioenvironmental factors and COVID-19, statistical analysis was performed using the MLR and GWR models. The GWR model has a consistent advantage in terms of parsimony, outperforms the classic regression model substantially [47, 48] and interact data from different platforms [49]. Both models require certain criteria to meet the requirements of assumptions such as linearity, normality, absence of multicollinearity, independence of observation, and heteroskedasticity [50, 51]. To obtain the assumption results, we used the Kolmogorov–Smirnov and Shapiro-Wilk test (normality), Durbin–Watson test (autocorrelation), variance inflation factor (VIF for multicollinearity) and scatter plot observations (for heteroskedasticity) [49]. The objective of tests is to guarantee that the regression model is unbiased and consistent and demonstrates estimation accuracy [52]. The advantages of the MLR and GWR models are they can be compared with each other, refer to multivariable regressions and have similar dataset testing criteria.

When the assumption tests were completed, the interactions between the variables can be revealed through MLR (Eq. 1) and GWR (Eq. 2). The independent variables in the two models have a significant effect if the F-count value is greater than the F-table value, and the determination rate of the independent variables can refer to the r-squared and adjusted r-squared values with *p*-values less than 0.05 with 95.0% confidence level [53]. The interactions between the variables can be determined partially through the Pearson correlation test, whilst the level of significance refers to the r-value, and *p*-value [54]. The spatial distribution of each variable is revealed through Moran’s I spatial autocorrelation [55] (Eq. 3). The significance of these statistical analyzes is divided into *p*-values of 0.05, 0.01, and 0.001. Especially for *p*-value of 0.05, the threshold is indicative because the confidence interval does not always overlap [56]:

1 $$Y=a+{b}_1{X}_1+{b}_2{X}_2\dots .+{b}_n{X}_n$$

2 $${Y}_i={\beta}_0\left({u}_i,{v}_i,\right)+{\beta}_k\left({u}_i,{v}_i,\right)\ {x}_{ik}+{\varepsilon}_i,$$

where *Y* is the dependent variable; *a* is constant; *b* is the slope of the line (regression coefficient); *X* is the independent variable; *Y*_*i*_ is the observed value of the dependent variable (located in *i*); *u*_*i*_*v*_*i*_ is the geographical location of *i*; *β*_*0*_*(u*_*i*_*v*_*i*_*)* is the predictor coefficient of k, which is located in *i*; and dan *ε*_*i*_ is the geographical constant.

3 $$I=\frac{{\sum\nolimits_{\text{i}=1}^\text{n}}{\sum\nolimits_{\text{j}=1}^\text{n}}[{\text{W}}_\text{ij}]\left(X_\text{i}-\overline{\mathrm{X}}\right)\left(X_\text{j}-\overline{\mathrm{X}}\right)}{\left({\sum\nolimits_{\text{i}=1}^\text{n}{\sum\nolimits_{\text{j}=1}^\text{n}}{\text{W}}_\text{ij}}\right){\sum\nolimits_{\text{i}=1}^\text{n}}{\left({\text{X}}_\text{i}-\overline{\mathrm{X}}\right)^2}},$$

where *I* is the Moran’s statistics value, *X*_*i*_ is a variable of *i*, *X*_*j*_ is a variable of *j* and *W*_*ij*_ is the weight influencing the spatial interaction of *i* and *j.*

The best model to reveal the spatial interactions refers to the lowest residual value, and the superiority of the GWR model over the MLR model can be viewed through the magnitude of the r-squared value [57]. In addition, the best model can refer to the value of deviance (Dev), degree of freedom (DoF), Dev per DoF (DDoF) and Akaike Information Criterion (AIC) as evidence of geographical weighting in accordance with the First Law of Geography or Tobler’s Law, stating that ‘Everything is related to everything else, but closer things are more related than distant things’ [58, 59]. To obtain geographical weighting, this study used a fixed Gaussian and bi-square adaptive kernel functions of GWR model [60]. The bandwidth value was obtained using the golden section search method. Meanwhile, the spatial variability of the model was based on positive or negative Dev, DoF, DDoF and AIC values [44, 61]. Compared with the MLR model, the spatial effect was feasible in the GWR model, as it considered the spatial location of the variables.

**Fig. 2:**
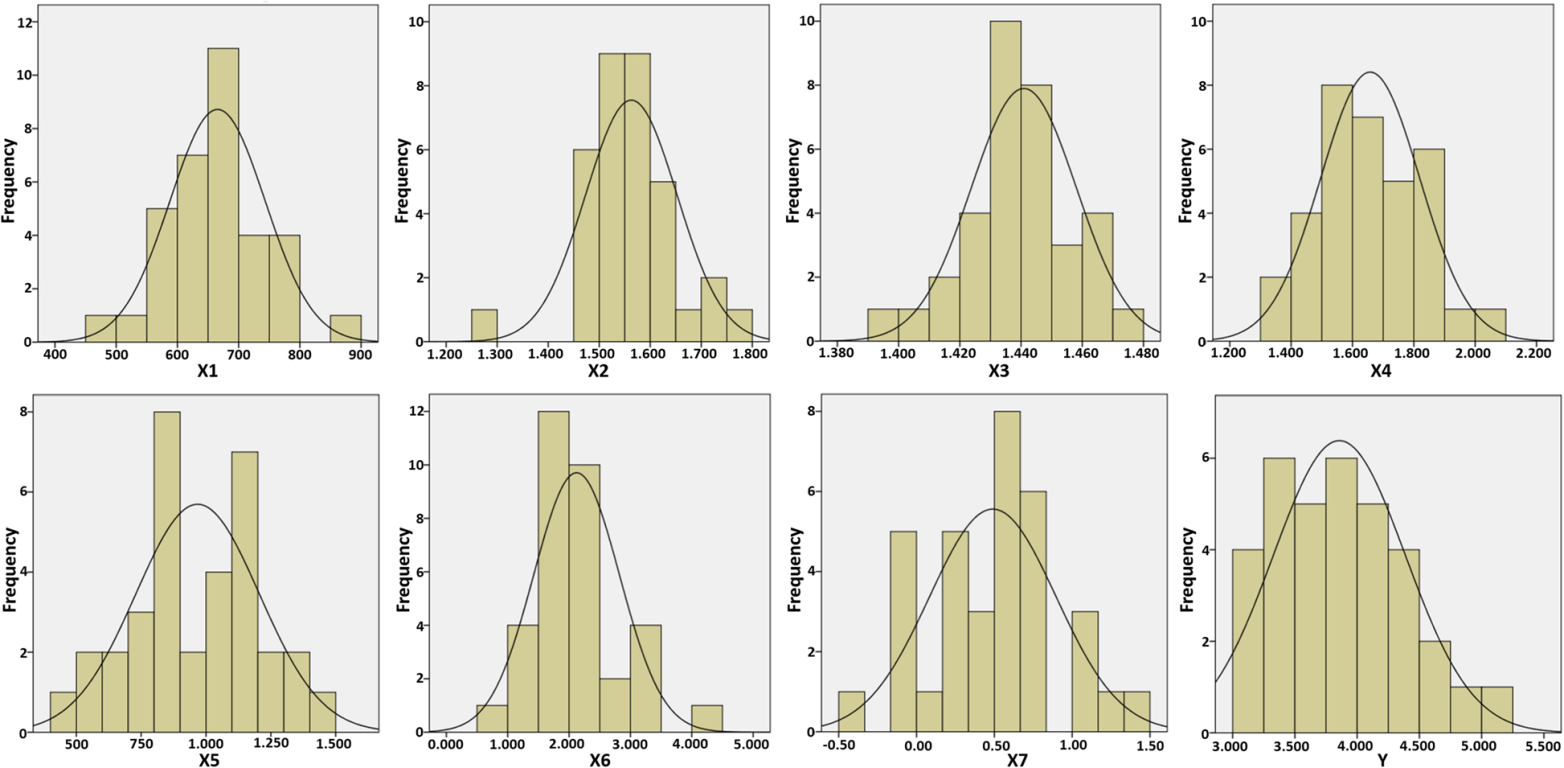
Histogram of independent and dependent variables

Before the results of the spatial interactions between the socioenvironmental factors and COVID-19 were discussed, the assumption tests were conducted for the MRL and GWR models to enable them to perform the appropriate statistical analysis and make determinations on the dependent variable [62–64]. The results of the normality test using the Kolmogorov–Smirnov (K-S) and Shapiro-Wilk (S-W) methods showed that all the variables had a normal distribution pattern with a *p*-value of > 0.05 (Table 1 and Fig. 2), which were suitable for the research with 34 to 50 sample data [65, 66]. Meanwhile, the autocorrelation test involving the independent variables showed that all the socioenvironmental factors were free, as the Durbin–Watson value reached 2022 (k = 7, *n* = 34) and met the distribution threshold.

**Table 1:** Normality test results

Parameter	X1	X2	X3	X4	X5	X6	X7	Y
**Test statistics**	0.124	0.101	0.098	0.104	0.088	0.130	0.083	0.080
***P*-value (K-S)**	0.200	0.200	0.200	0.200	0.200	0.158	0.200	0.200
***P*-value (S-W)**	0.537	0.094	0.780	0.572	0.829	0.105	0.790	0.404

**Fig. 3:**
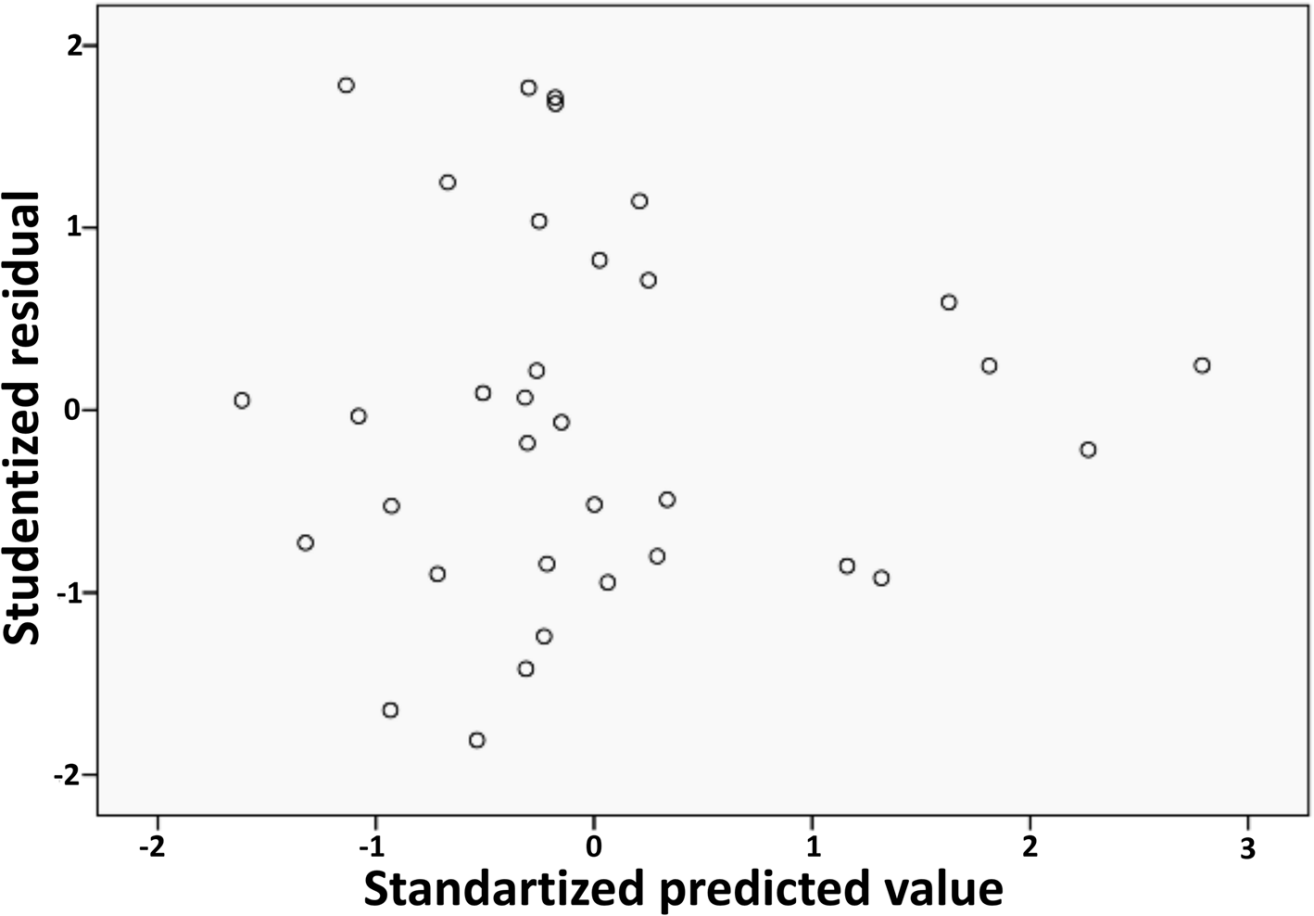
Scatter plot for heteroscedasticity observation

In terms of multicollinearity, all the variables achieved the required VIF value of < 10.00 as shown in Table 2. We also estimate that multicollinearity will not interfere with the statistical model significantly because the tolerance value is more than 0.1 [51, 52], although, Table 9 shows there are correlations between the independent variables. The fulfilment of the linear regression assumptions was followed by the heteroscedasticity test, in which the scatter plot showed even distribution points and did not form a wave pattern (homoscedasticity) (Fig. 3) [67], this result is reinforced by the F-statistic of 1.06 from Glejser-test with a *p*-value of 0.42 > 0.05 [68, 69]. Thus, all the classical assumptions for the MLR and GWR analyses were declared fulfilled.

**Table 2:** VIF as the multicollinearity indicator

Independent Variables		Collinearity Statistics	
		Tolerance	VIF
Internet Development Index (IDI)	X_1_	0.13	7.91
Literacy Index (LI)	X_2_	0.18	5.70
Average temperature (AT)	X_3_	0.91	1.10
Urban Index (UI)	X_4_	0.24	4.11
Poverty Rate (PR)	X_5_	0.57	1.74
Population Density (PD)	X_6_	0.26	3.85
Commuter Worker rate (CW)	X_7_	0.28	3.55

## Results

COVID-19 has spread to many countries around the world, including tropical regions. Since it was first announced by the president of the Republic of Indonesia in 2020, COVID-19 has infected hundreds of thousands of people in the country [70]. At the end of December 2020, the number of confirmed cases in Indonesia reached more than 743,000, with COVID-19 spreading across the country. This number is expected to increase if the government and society continue to lack common awareness, especially regarding the implementation of health protocols [71]. The findings of this study reveal that the spread of COVID-19 in Indonesia can be explained by pandemic developments, socioenvironmental factors and the interplay between the socioenvironmental factors and COVID-19.

### COVID-19 pandemic

**Fig. 4:**
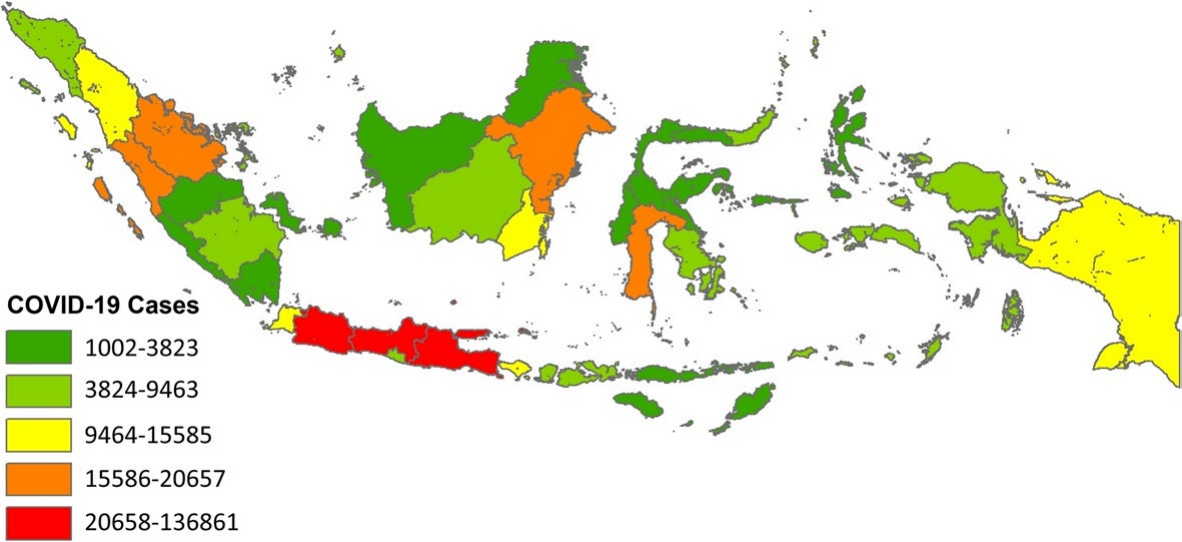
Distribution of confirmed COVID-19 cases until November 2020

The number of confirmed positive COVID-19 cases in Indonesia has exceeded 538,883. The fatality rate is 3.1%, and the number of recovered cases is 450,518, based on the 30 November 2020 records of the Indonesian Ministry of Health. However, the number of new cases continues to increase, with the high daily record exceeding the daily patient recovery rate [32, 72]. The distribution of COVID-19 cases in the country is diverse, based on the spatial distribution in each province. DKI Jakarta recorded the highest number of confirmed cases, followed by East Java, Central Java and West Java (Fig. 4). The four provinces account for 57% of the total number of confirmed cases in the country, thereby causing health facilities (hospitals) to nearly exceed their capacity, especially as numerous health workers have become infected or died [73].

The average number of confirmed COVID-19 cases in each province has reached 15,850, whereas the range between the regions (maximum and minimum) could reach 121,012 cases. This fairly high difference in range indicates the clustered pattern of the confirmed cases in several regions. COVID-19 in Indonesia is concentrated in Java, as indicated by a z-score of 2.24. This clustering tendency shows a similar trend to deaths by SARS-CoV-2 caused either by pneumonia or the weakening of the immune system, which can aggravate the severity of comorbidities [74]. Nevertheless, several provinces outside Java Island, such as South Celebes, Bali, and North Sumatra, have high confirmed COVID-19 cases. Based on the spatial distribution of COVID-19 cases, without the existence of policies in each province implementing health protocols or limiting intraregional and interregional movements, the number of positive cases will continue to increase owing to the emergence of new clusters triggering local transmission [75].

### Socioenvironmental factors

A disease outbreak caused by a virus or other microorganisms requires ideal conditions for transmission to continue and the virus to acquire new vectors [76]. The COVID-19 pandemic resulted from interactions between physical and socioeconomic environments known as socioenvironmental factors, and the complexity of the interactions between these factors causes disease transmission characteristics to either differ or become similar in different regions [77]. The socioenvironmental factors used in this study as the main indicators of regional development are the IDI (X_1_) and LI (X_2_). Since the introduction of the Internet in Indonesia in the 1990s, its development has continued to improve, with residents currently enjoying 4G services on their smartphones from various cellular operators [78].

Internet access has become a necessity in education, economic transactions, entertainment, and communication. The average IDI in Indonesia is 4.70, with DKI Jakarta having the highest score (Table 3). However, a high IDI value does not necessarily indicate a high literacy level, which is crucial during a pandemic, as the ability to understand and obtain information is necessary for the successful implementation of health protocol programs, considering Indonesia’s low literacy rates [79]. The distribution of the LI value of each province shows a gap between the index values, with a fairly high range, and the average value. Moreover, in COVID-19 emergency response, literacy does not consistency lead to controlled action, especially if personal conditions and the surrounding environment encourage the neglect of the virus [80]. In terms of disasters in general, COVID-19 has an impact on economically weak communities (PR; X_5_) with residents with no savings [81]. This outcome forces such communities to ignore preventive measures, such as movement control and the limitation of interactions, owing to the residents’ financial demands to sustain their primary needs.

**Table 3:** Central tendency of socioenvironmental factors

Parameters	X_**1**_	X_**2**_	X_**3**_	X_**4**_	X_**5**_	X_**6**_	X7
Maximum	7.61	58.16	30.00	100.00	27.55	15,900.00	21.90
Minimum	2.95	19.90	25.10	23.00	3.15	9.00	0.40
Average	4.70	37.32	27.62	48.66	10.70	741.38	4.65
Range	4.66	38.26	4.90	77.00	24.40	15,891.00	21.50
Standard deviation (SD)	0.87	7.89	1.09	18.75	5.85	2708.90	4.68
N	34	34	34	34	34	34	34

Pressure from the surrounding environment will increase, especially for people living in urban areas. The livelihood base in the manufacturing and service sectors forces residents to interact intensively with one another, thereby making virus transmission easy; hence, this phenomenon can be understood with the UI (X_4_) [82]. The results show that the UI value is centered in Java Island, where nearly all major cities developed hundreds of years ago, though efforts for the equitable distribution and development of new growth centers are currently being discussed, including the relocation of the country’s capital to Kalimantan (Borneo) [83]. In addition, the manufacturing and service sectors are mostly located in urban areas, thereby entailing the daily shuttle or commute of workers (X_7_) from surrounding suburbs or rural areas, especially when the new normal policy is perceived by industries as permission to reopen their business. Currently, the highest number of CWs is recorded in DKI Jakarta, Banten, DI Yogyakarta and West Java, which have major growth centers [84]. Workers who do not want to commute choose to settle near their workplace, thereby increasing the PD (X_6_) of the province and the industrial-based sector [85]. Currently, Java has the highest population concentration and density in Indonesia.

**Fig. 5:**
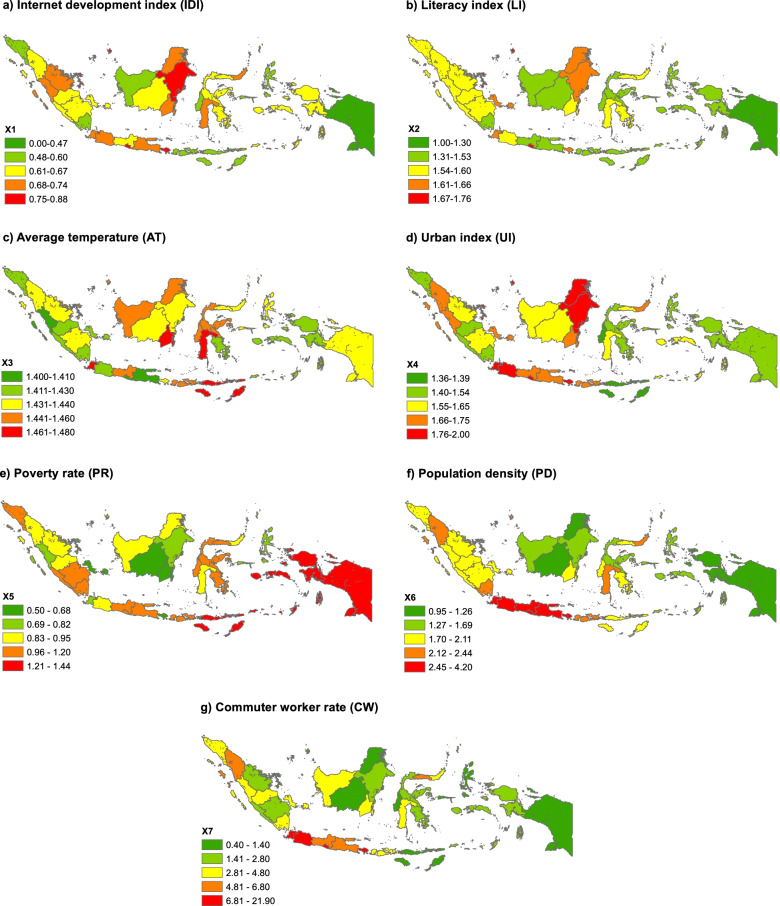
Distribution of socioenvironmental factors

Faced with high PD and challenges to improve economic conditions and health, the Indonesian government took risks, which further increased the number of confirmed COVID-19 cases in the country. As a tropical country, Indonesia has limiting factors for the growth and transmission of the virus, that is, ultraviolet radiation from the sun throughout the year. Ultraviolet radiation has very high prevalence with the outdoor AT value (X_3_) in the tropics [86]. Therefore, the temperature is assumed to be one of the controllers of COVID-19 in tropical environments, where the average temperature of every province of Indonesia can reach up to 27.62 °C. The differences in the spatial distribution and variation of the socioenvironmental factors are presented in Fig. 5.

The analysis shows that the socioenvironmental factors result in different and unequal spatial distribution characteristics, that is, either a centralized or dispersed pattern. Clustered distribution occurs significantly in the UI, PD and CW rate, whereas random distribution occurs in the IDI, LI, AT and PR (Table 4). Although Indonesia is a tropical country with consistent average temperatures that do not exhibit high fluctuations, the spatial autocorrelation results show random characteristics in the spatial distribution of the temperature of the different provinces. The phenomenon showing the random distribution of air temperature seems reasonable owing to the local and monsoon influences on the local climate of the provinces, which are spread across islands with a large landmass and tiny islands exposed predominantly to the influence of the sea [87, 88]. The difference in the spatial distribution of the socioenvironmental factors may potentially contribute to the increase in the number of COVID-19 cases. Meanwhile, the IDI, LI and PR variables demonstrate random distribution, which indicates unbalanced national development efforts and socioeconomic threats endangering the residents of each province.

**Table 4:** Spatial autocorrelation of socioenvironmental factors

Variable	Moran’s Index	Z-score	***P***-value	Status
Internet Development Index (IDI)	0.11	1.50	0.13	Random
Literacy Index (LI)	0.04	0.75	0.45	Random
Average Temperature (AT)	0.04	0.69	0.49	Random
Urban Index (UI)	0.27	3.07	0.00	Clustered
Povertry Rate (PR)	0.12	1.55	0.12	Random
Population Density (PD)	0.74	7.98	0.00	Clustered
Commuter Worker rate (CW)	0.71	8.05	0.00	Clustered

### Spatial interactions between socioenvironmental factors and COVID-19

The interactions between the socioenvironmental factors and COVID-19 in Indonesia are significant, with *p*-values of < 0.01, based on the MLR and GWR models. Based on the MLR model, the simultaneous interactions between the independent and dependent variables show an r-squared value of 0.68, which means that the socioenvironmental factors can explain 68% of the confirmed COVID-19 cases. However, when geographical locations are involved, the r-squared value of this interaction model increases to 0.70 (Table 5). The GWR model shows that the COVID-19 cases are influenced by the independent variables located near one another. If some of the provinces have similar socioenvironmental characteristics, then they will have the same high number of confirmed COVID-19 cases (accumulative) as the consequence of the GWR model [89, 90]. Both models have an F-count value higher than the F-table value (7.74 > 3.98).

**Table 5:** Comparison of simultaneous influence between MLR and GWR models

Model	R	r-squared	Adj. r-squared	SE	Sum of squares (SS)	F
MLR	0.82	0.68	0.59	0.34	3.00	7.74^***^
GWR	0.84	0.70	0.56	0.35	2.81	

^***^p-value < 0.001

To determine whether spatial interactions produce an improved model, Table 6 shows the improvement in the GWR and MLR models. The suitability of the GWR model is better than that of the MLR model, and the GWR model has lower Dev, SS and DoF values and is reinforced by better mean square and − 2 log-likelihood values despite having the opposite AIC parameter. Differences in one parameter will not eliminate the advantages of a statistical model if the other parameters support the model [91, 92]. Moreover, in this case, the AIC value exhibits only a small difference of 0.65, which could occur owing to the differences in the coefficient value of each independent variable.

**Table 6:** Comparison between regression models and their variances

Model	Dev	DoF	Dev per DoF	AIC	Mean square	−2 log-likelihood
MLR	3.01	26.00	0.12	32.05	0.90	14.05
GWR	2.81	23.47	0.12	32.70	0.12	11.71

**Fig. 6:**
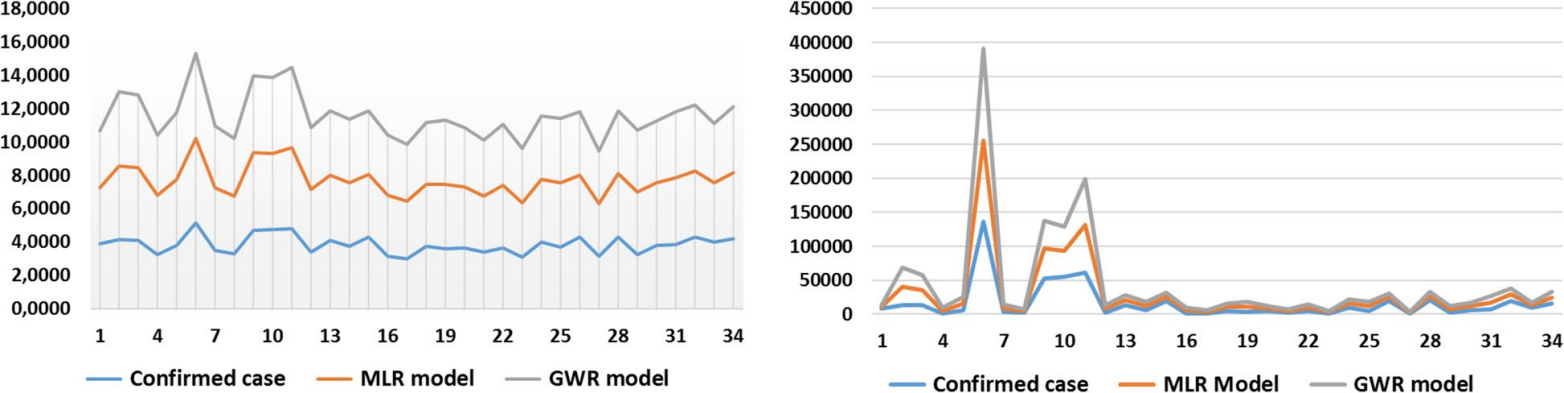
Comparison between confirmed COVID-19 cases, MRL model and GWR model

The GWR model is selected as the best option to reveal the interactions between the socioenvironmental factors and COVID-19 in Indonesia based on the SD of the coefficient of variation of the independent variables (Table 7 and Fig. 6). The GWR model has a low deviation, which means that the independent variables can estimate effectively and closely to the average value [93]. Table 8 also shows that the IDI, UI and PD contribute positively to the confirmed COVID-19 cases. However, the LI, AT, PR, and CW rate of each province have opposite contributions. In Indonesia, the more developed the area in terms of the urbanised environment, the higher its case contribution to the pandemic. By contrast, the government can slow the spread of COVID-19 by improving and raising literacy, as numerous residents do not believe in SARS-CoV-2 and vaccines (antivaccine groups) [71, 94, 95]. In addition, this interaction model indicates that AT plays a significant role in pandemic transmission, as seen in the spike in cases during the wet season, when temperatures are lower and more humid than usual [96].

**Table 7:** Coefficient of independent variables in MLR and GWR models

Independent Variables		MLR Model		GWR Model	
		Β	SD	Β	SD
Constant		12.65^***^	–	11.99^***^	0.83
Internet Development Index (IDI)	X_1_	6.40^***^	0.94	7.01^***^	0.25
Literacy Index (LI)	X_2_	−6.89^***^	−1.17	−6.99^**^	0.21
Average Temperature (AT)	X_3_	−3.28	−0.11	−2.83	0.68
Urban Index (UI)	X_4_	1.01	0.31	0.86	0.20
Poverty Rate (PR)	X_5_	−0.16	−0.07	−0.16	0.02
Population Density (PD)	X_6_	0.46^*^	0.61	0.46^*^	0.01
Commuter Worker rate (CW)	X_7_	−0.11	−0.08	−0.11	0.02

^***^p-value < 0.001, ^**^p-value < 0.01 ^*^p-value < 0.05 with 95.0% confidence level

**Table 8:** Spatial autocorrelation of actual cases and regression models

COVID-19	Moran’s Index	Z-score	***P***-value	Status
Actual confirmed cases	0.19	2.24	0.02	Clustered
MLR model	0.56	6.07	0.00	Clustered
GWR model	0.54	6.00	0.00	Clustered

## Conclusion

COVID-19 in Indonesia has a spatial relationship with various socioenvironmental factors through the MLR and GWR models. The GWR model is better than the MLR model for explaining the spatial effects of the interactions between the independent and dependent variables, with an r-squared value of 0.70. This study shows that the IDI, LI, AT, UI, PR, PD and CW rate simultaneously influence the increasing confirmed COVID-19 cases in the 34 provinces of Indonesia. When referring to the local r-squared value of the GWR model, the highest simultaneous interactions occur in the three provinces of East Nusa Tenggara, West Nusa Tenggara and Bali. The link between socioenvironmental factors and COVID-19 should be emphasised in mitigation plans to control the transmission rate in areas with high PD, urbanized spaces, and CW, this effort will prevent health facilities from collapsing. However, the contribution of socioenvironmental factors to the pandemic requires further research to reveal other factors and improve management. Furthermore, this research has several limitations. For example, it does not examine city or regency region II levels in the context of Indonesian political-administration after the regional autonomous. Future studies should add other independent variables, such as public perceptions and attitudes, policy implementation, access to and quality of health services and the effectiveness of health communication. Finally, understanding of the spread of diseases can also be improved to save lives.

## Discussion

**Fig. 7:**
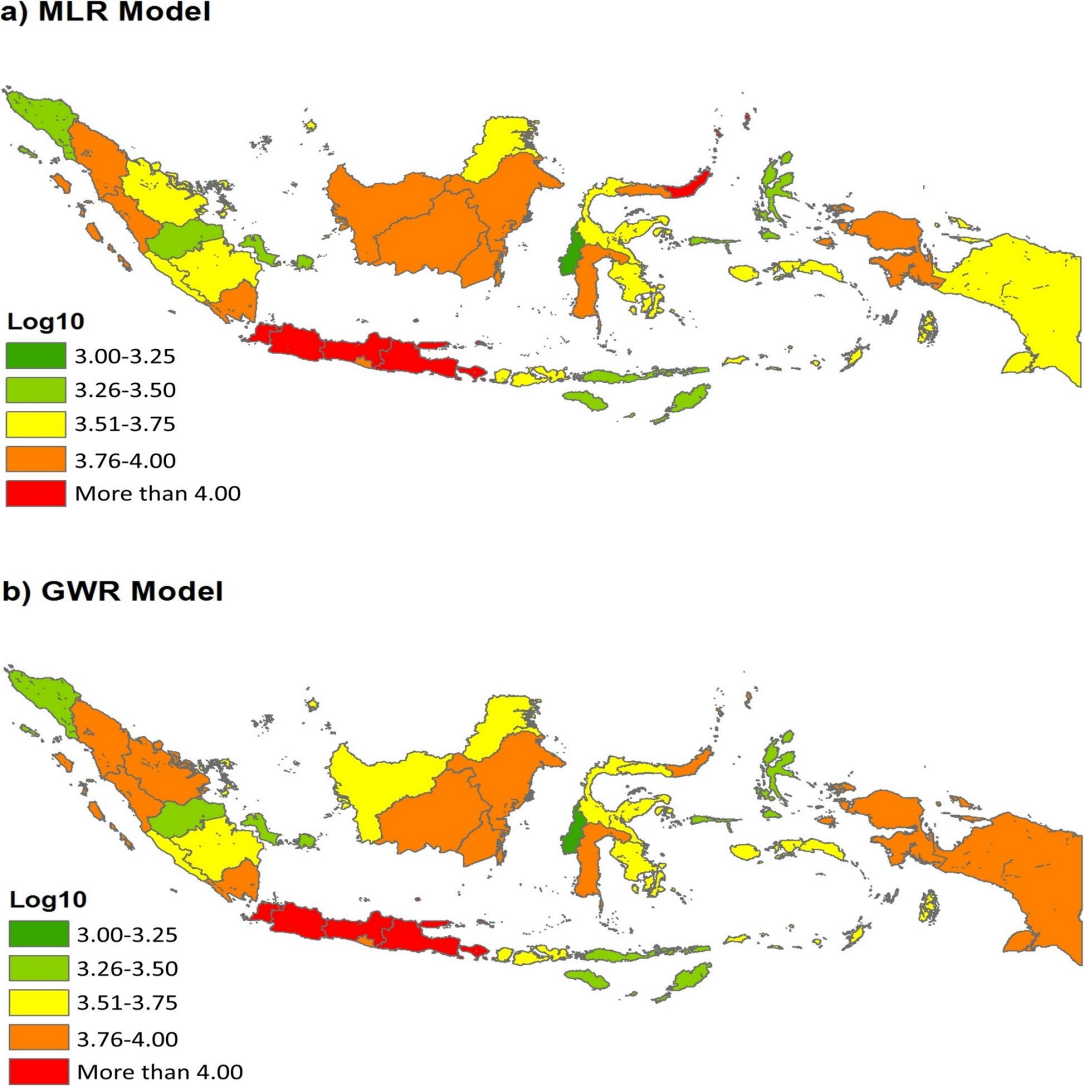
Spatial distribution of **a**) MRL and **b**) GWR models

**Fig. 8:**
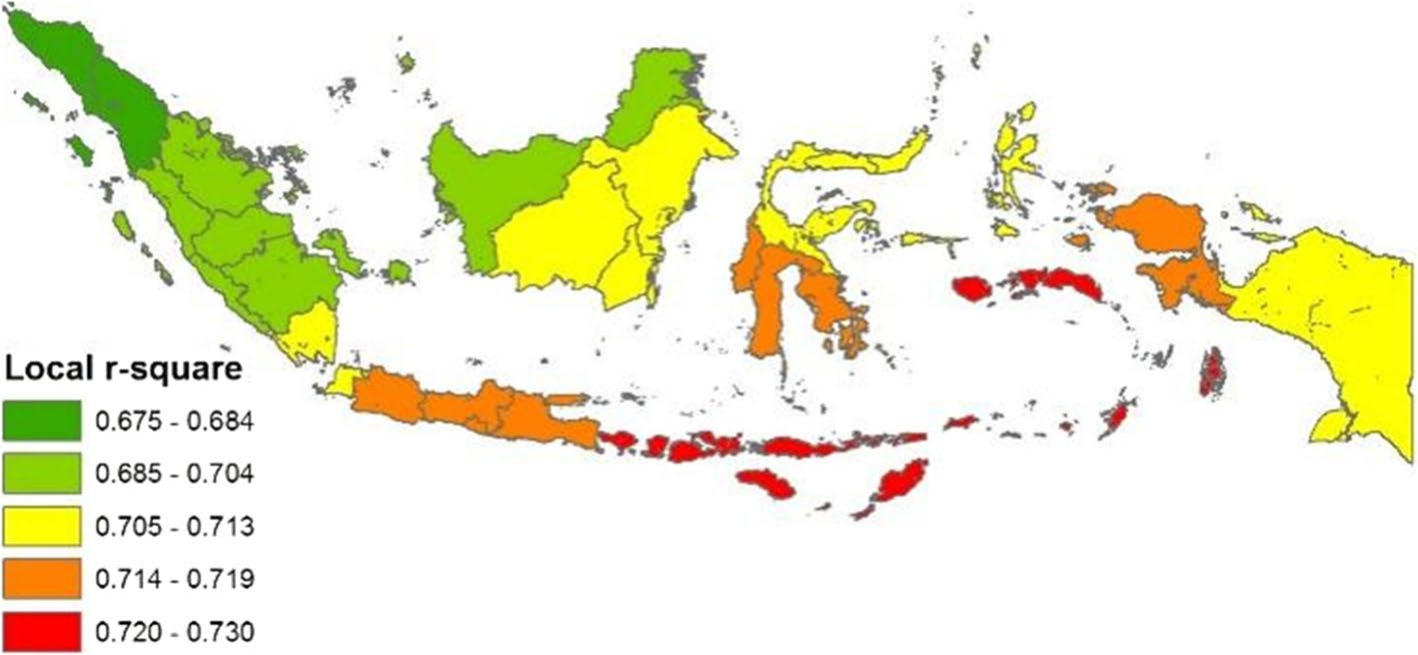
Local r-squared distribution based on GWR model

The actual situation of the COVID-19 pandemic in Indonesia has a central distribution pattern. The interactions from the MLR and GWR models also produced similar results, that is, a clustered pattern (Table 8). The COVID-19 pandemic and socioenvironmental factors (simultaneously) have similar patterns when compared with the spreading trend of COVID-19 in Figs. 7 and 8. The spatial interactions differ only in a few regions, such as North Celebes, Riau, West Borneo and Gorontalo. The spatial interaction models also jointly confirm Java as the centre of the pandemic in Indonesia, followed by Bali, North Sumatra and South Celebes. If not managed wisely, the COVID-19 pandemic may become an epidemic in the country, such as malaria, dengue hemorrhagic fever and tuberculosis [97–99].

In addition to the interactions between the socioenvironmental factors and COVID-19, understanding the disease through the partial correlation between the independent variables is necessary [100–102]. Table 9 shows a very high correlation between the socioenvironmental factors, such as the IDI with the LI and UI, which indicates harmony in regional development rates, including increased access to health services. The government and other stakeholders should focus on PD, which has a high correlation with the UI and a very high correlation with the CW rate and should be controlled in the context of outbreak management. Java, as the center of economic activities and the most populated island in Indonesia, is home to megapolitans with interprovincial influence, such as Greater Jakarta, Bandung Raya, and Surabaya (Gerbang-Kertosusila), where daily migration of commuters is common in these areas [103]. Moreover, the spatial interaction model in Eqs. 4 and 5 indicates that commutation level is a potential controller, because the UI is an uncontrolled factor in the short term.

4 $$\mathrm{Y}=12.65+6.40\ \left({\mathrm{X}}_1\right)-6.89\ \left({\mathrm{X}}_2\right)-3.28\ \left({\mathrm{X}}_3\right)+1.01\ \left({\mathrm{X}}_4\right)-0.16\ \left({\mathrm{X}}_5\right)+0.46\ \left({\mathrm{X}}_6\right)-0.11\ \left({\mathrm{X}}_7\right)$$

5 $$\mathrm{Y}=11.99+7.01\ \left({\mathrm{X}}_1\right)-6.99\ \left({\mathrm{X}}_2\right)-2.83\ \left({\mathrm{X}}_3\right)+0.86\ \left({\mathrm{X}}_4\right)-0.16\ \left({\mathrm{X}}_5\right)+0.46\ \left({\mathrm{X}}_6\right)-0.11\ \left({\mathrm{X}}_7\right)$$

**Table 9:** Partial correlation between independent variables

Variable		X_**1**_	X_**2**_	X_**3**_	X_**4**_	X_**5**_	X_**6**_	X_**7**_
Internet Development Index (IDI)	X_1_	1	0.90^***^	−0.03	0.85^***^	−0.60	0.55^**^	0.51^**^
Literacy Index (LI)	X_2_	0.90^***^	1	−0.03	0.77^***^	−0.58^***^	0.56^**^	0.46^**^
Average Temperature (AT)	X_3_	−0.03	−0.03	1	−0.04	− 0.18	0.06	− 0.03
Urban Index (UI)	X_4_	0.85^***^	0.77^***^	−0.04	1	−0.55^**^	0.59^***^	0.58^***^
Poverty Rate (PR)	X_5_	−0.60^***^	−0.58^***^	− 0.18	−0.55^**^	1	−0.29	− 0.25
Population Density (PD)	X_6_	0.55^**^	0.56^**^	0.06	0.59^***^	−0.29	1	0.83^***^
Commuter Worker rate (CW)	X_7_	0.51^**^	0.46^**^	−0.03	0.58^***^	−0.25	0.83^***^	1

^***^p-value < 0.001, ^**^p-value < 0.01

The socioenvironmental factors have a distribution pattern similar to that of COVID-19, which illustrates the location suitability of SARS-CoV-2 to infect people as a viral vector [81, 104–106]. Efforts to understand the interactions simultaneously or partially can be meaningful for effective and efficient decision making in handling COVID-19 in Indonesia, because we are confronted with viral mutations that can increase the disease transmission and reduce the effectiveness of vaccines e.g. Alpha, Beta, Gamma, Gamma, and Delta variants [107]. This step could be better than simply referring to pandemic prediction models, which only consider virus transmission based on increasing rates of daily cases until the saturation point is reached and mostly miss, especially those created during the early stage of the pandemic [108–110] owing to insufficient attention to overfitting. The GWR spatial interaction model can serve as input for the development of a pandemic model for Indonesia, which can determine up to 70% of the spreads of COVID-19 pandemic cases.

## Data Availability

The datasets used and analysed in this study are available from the corresponding author on reasonable request.
